# Head and neck cancer: metronomic chemotherapy

**DOI:** 10.1186/s12885-015-1705-z

**Published:** 2015-10-12

**Authors:** Francesca De Felice, Daniela Musio, Vincenzo Tombolini

**Affiliations:** 1 Department of Radiotherapy, Policlinico Umberto I “Sapienza” University of Rome, Viale Regina Elena 326, 00161 Rome, Italy; 2 Spencer-Lorillard Foundation, Viale Regina Elena 262, Rome, Italy

**Keywords:** Metronomic chemotherapy, Low dose chemotherapy, Head neck cancer

## Abstract

In the era of personalized medicine, head and neck squamous cell carcinoma (HNSCC) represents a critical oncologic topic. Conventional chemotherapy regimens consist of drugs administration in cycles near or at the maximum tolerated dose (MDT), followed by a long drug-free period to permit the patient to recover from acute toxicities. Despite this strategy is successful in controlling the cancer process at the beginning, a significant number of HNSCC patients tend to recurred or progress, especially those patients with locally advanced or metastatic disease. The repertoire of drugs directed against tumor cells has greatly increased and metronomic chemotherapy (MC) could be an effective treatment option.

It is the purpose of this article to review the concept of MC and describe its potential use in HNSCC. We provide an update of ongoing progress and current challenges related to this issue.

## Background

In 2015, there will be an estimated 59340 new cases of head and neck squamous cell carcinoma (HNSCC) in the United States [1]. Despite advances in surgery (S), radiotherapy (RT) and chemotherapy (CHT), the survival of patients with HNSCC has not improved significantly over the past decades. The main reason for treatment failure is the development of loco-regional recurrences and/or metastasis, especially in patients with locally advanced disease. Based on Extreme trial results, platinum-fluorouracil chemotherapy plus cetuximab is nowadays considered the first-line standard treatment, due to the significant improvement in median overall survival (OS) and progression free survival (PFS) compared with CHT alone (10.1 and 5.6 months versus 7.4 and 3.3 months, respectively) [2]. However the response rate is still low (36 %) [2].

Metronomic chemotherapy (MC) is an emerging therapeutic option in clinical oncology and it may prove useful at least in metastatic HNSCC patients. To develop rational therapeutic strategies, it is important to identify molecular targets that are linked to the pathogenesis of HNSCC. This article provides an overview of MC and the rationale for investigating whether MC could be considered as a valid strategy option in the management of HNSCC.

A literature search of Medline database was performed using the following search terms “metronomic chemothereapy”, “head neck”, “solid tumor” and “continuous low dose”. English-literature was considered. Additional references were selected from relevant papers. There is limited literature regarding MC in HNSCC. To identify extra data on HNSCC, solid tumor studies were firstly considered to verify whether it was possible to extrapolate further relevant information in HNSCC cases. Moreover a search of the clinical.gov database was conducted to identify ongoing clinical trials. Search strategy was performed up to May 2015.

### The rationale

In recurrent and/or metastatic HNSCC patients, survival rates are low and several CHT regimens are nowadays available, after failure of first-line platinum-based regimen, to improve cancer treatment. Successive lines of CHT consist of taxanes alone or in combination with cetuximab, capecitabine and metothrexate, depending on previously given CHT regimens [3]. The goal is to affect different molecular targets and cell metabolism pathways.

Considering the antiangiogenic activity of cytotoxic agents, MC could be proposed to patients with HNSCC progression, resistant to other CHT lines or with impaired general conditions. Taxanes are cytotoxic agents active against endothelial cells at low-doses; cisplatin blocks specific steps of angiogenic cascade; methotrexate interferes with endothelial cell functions without cytotoxic effect [4].

In general, MC increased patient outcomes and showed clinical benefit (from 30 to 93 %) in other cancer setting, including advanced breast cancer, recurrent ovarian cancer and advanced cancer of various tumor types [5]. The wide range of response rate seems to be ascribed to the vast patient heterogeneity. However, both survival and clinical benefits could be theoretically transposed to HNSCC and deserve further studies. MC could induce tumor response in those cases that showed acquired drug resistance after conventional CHT and its tolerability should compare favorably with other CHT lines administered [6]. By reducing the time between administrations, the purpose is to prevent tumor cells regrowth and minimize the possibility of CHT resistance.

### Overview

The term “metronomic” derives from the musical device “metronome” that produces regular and metrical ticks that represent a regular aural pulse. In the same way, MC is the regular administration of CHT that results in a constant low blood level of the drug. Thus MC does not refer to the mechanism of action of the antineoplastic drug, but it reflects the frequent administration of low doses (1/10^th^–1/3^rd^ of the maximum tolerated dose [MTD]) of drugs, at shorter intervals without interruption. It represents a different philosophy from the conventional CHT administration that is based on the MTD, the highest dose of a treatment with acceptable toxicity able to kill as many tumor cells as possible.

MC exerts its anti-cancer activity by inhibiting tumor angiogenesis, stimulating anticancer immune response and inducing tumor dormancy.

Angiogenesis, the growth of new blood vessels, is associated to tumor growth and metastasis. Due to the faster cell proliferation, as well as the presence of immature endothelial cells in the basement membrane of new vessel walls and the lack of both innervation and collateral supply, the vascular endothelium is an attractive vulnerable element in the tumor [7]. Thus drugs that inhibit angiogenesis process represent a strategy for stopping cancer, especially by affecting the regrowth of intratumoral vascular endothelial cells. At the beginning, MC efficacy was exclusively relied on its anti-angiogenesis mechanism and several angiogenesis inhibitors or anti-angiogenesis drugs were developed and investigated. The vascular endothelial growth factor (VEGF) was probably the most important angiogenesis stimulator and was believed to play a key role in the neovascularization of human tumors. It has been suggested that both tumor cells and their supporting endothelial cells represent the main target site [8]. The MC antiangiogenic effect was demonstrated by Browder and Klement in their preclinical studies of experimental cancer conducted in vitro and in vivo [6, 9]. Browder et al. showed that cyclophosphamide-based MC provided sustained apoptosis of endothelial cells, resulting in eradication of Lewis lung carcinoma and L1210 leukemia [6]. Klement et al. revealed that low doses of vinblastine in association with anti-VEGF resulted in full regressions of large established tumors, without increasing toxicity [9].

MC induced also important immunomodulatory effects. The activation of both innate and adaptive immune system is mediated by some drugs, including taxanes and cyclophosphamide. It has been demonstrated that MC selectively induced reduction of circulating regulatory T cells and subsequent reduction of their inhibitory functions on antigen-specific immune response [10].

Induction of tumor dormancy is nowadays considered a potential mechanism of MC. Dormancy is defined as a pause in cancer progression. There are three mechanisms of cancer dormancy including angiogenic dormancy (inability of tumor cells to recruit blood vessels), cellular dormancy (tumor cells in G0-G1 arrest) and immunosurveillance (prevent residual tumor cells expansion) [11]. Therefore, by inhibiting angiogenesis and controlling immune system, MC can promote tumor dormancy.

### Preclinical trials

Considering that a considerable percentage of HNSCC over-express anti-apoptotic Bcl-2 proteins and that high levels of Bcl-2 correlates with resistance to platinum-based CHT and thus with a poor prognosis, Bcl-2 pathway has become an attractive target for investigating the potential therapeutic effects of new drugs [12, 13]. The Bcl-2 protein family has four Bcl-2 homology (BH) domains (BH1, BH2, BH3 and BH4) that are involved in cellular activities.

Imai et al. have demonstrated that MC, based on BH3-mimetic drug (AT101), in association with taxotere decreased both tumor mitotic index and microvessel density and increased survival of mice bearing HNSCC xenografts [14]. Authors evaluated the combination AT101/taxotere on the survival of endothelial cells and HNSCC also in vitro. They observed an additive toxicity for endothelial cells and a synergistic toxicity for tumor cells. Authors concluded that, based on these results, HNSCC patients might benefit from this MC regimen.

Same conclusions were proposed by Zeitlin et al. [15]. They investigated the effect of metronomic TW-37, an inhibitor drug that occupies the BH3 domain, in combination with RT in vitro and in xenograft models of HNSCC. The study showed that MC potentiates the RT anti-tumor effects.

### Clinical trials

The number of clinical trials of MC in HNSCC is still very limited, but results are promising (Table 1).

**Table 1:** Metronomic chemotherapy clinical trials in HNSCC patients

Author	Year	Study design	Patients (n)	Protocol (n patients)	Results
Patil et al. [16]	2015	phase II	110	celecoxib + methotrexate (57); cisplatinum (53)	OS 101 vs 66 days*; PFS 249 vs 152 days*
Pai et al. [17]	2013	retrospective	64	celecoxib + methotrexate (32); no MC (32)	2-year DFS 94.6 % vs 75.4 %*
Penel et al. [18]	2010	randomised	88	cyclophosphamide (44); megestrol acetate (44)	2-month PFS 20.5 % vs 9 %; median OS 195 vs 144 days

*n* number, *OS* overall survival, *PFS* progression free survival, *MC* metronomic chemotherapy, *DFS* disease free survival

*: *p*-value < 0.05

Patil et al. [16] believe that oral MC may be safer and more effective in patients with metastatic, relapsed or inoperable HNSCC. In a phase II trial, they randomized a total of 110 patients to oral MC (57 patients; daily celecoxib 200 mg × 2 and weekly methotrexate 15 mg/m^2^) versus platinum-based CHT (53 patients; cisplatinum 75 mg/m^2^ given 3 weekly). Overall survival, as well as progression free survival were significantly increased in MC patients compared to CHT arm (101 versus 66 days, *p* = 0.014 and 249 versus 152 days, *p* = 0.02, respectively). One limit of this trial is that the control group utilized only cisplatinum, which may have resulted in a worst outcome. Authors motivate this protocol due to cetuximab financial constrains.

Pai et al. [17] described their experience with MC in 32 locally advanced oral cancer patients, having a waiting period for surgery > 3 weeks. It was a retrospective chart analysis and MC was started while patients were awaiting surgery and continued during the perioperative period and after the adjuvant therapy. MC consisted of oral methotrexate 15 mg/m^2^ once a week and oral celecoxib 200 mg twice daily. Outcomes of these patients were compared with 32 stage-matched controls with similar waiting periods. In those patients who received at least 3 months of MC in the adjuvant setting, the 2-year DFS showed a statistically significant improvement compared to the control group (94.6 % vs 75.4 %, *p*-value 0.03).

Recently in conjunction with the 2015 ASCO Annual Meeting, Mateen et al. [18] have presented their experience with MC in recurrent HNSCC. A total of 72 patients were enrolled and were prescribed oral methotrexate 2.5 mg twice in a week and capecitabine 500 mg twice a day, for at least 6 months. Two-year progression-free survival and overall survival were 18 and 40 % respectively. Based on these results, authors concluded that MC is a valid option treatment in this setting of patients. Otherwise full text paper is still not available, thus results are not definitive.

To our knowledge, at present, no more published clinical trials on MC in HNSCC population are available. Consequently we have tried to collect HNSCC data from studies that have analyzed MC in solid tumors.

In a multicentre randomized phase II trial, Penel et al. have evaluated the safety and the efficacy of MC versus megesterol acetate in 88 patients (of whom 8 HNSCC cases) with progressive disease [19]. Eligible patients had a good PS and had exhausted all validated therapies under standard care. Results were favorable as reflected from the median overall survival (195 days versus 144 days in the MC arm and magestrol acetate arm, respectively).

Briasoulis et al. enrolled 62 patients with advanced refractory cancer to obtain preliminary data on efficacy of MC vinorelbine [20]. Of the entire cohort, only one HNSCC patient was recorded. Clinical response was documented in 8 patients and 32 % of patients experienced disease stability for minimum 6 months.

But, both studies suffered from patients heterogeneity and insufficient details of HNSCC patients, and therefore cannot provide additional support for the implementation of MC in HNSCC treatment.

### Ongoing trials

There are two ongoing clinical research studies testing MC in head and neck cancer (https://clinicaltrials.gov). The “Metronomic Chemotherapy With Tegafur/Uracil for Patients With Locally Advanced (Stage III ~ IVB) Head and Neck Squamous Cell Carcinoma (HNSCC)” (ClinicalTrials.gov Identifier: NCT00855881) is a trial conducted at the Mackay Memorial Hospital, Taipei, Taiwan. It is a phase II trial in which patients with histologically confirmed non-nasopharyngeal HNSCC are treated with tegafur-uracil, an oral fluoropyrimidine prodrug, for 1 year after complete response to previous treatment. Primary outcome is to evaluate the 2-year relapse free survival. This study is currently recruiting participants and preliminary results on primary outcome measures are still not available.

The Masonic Cancer Center, University of Minnesota has proposed a phase II study (ClinicalTrials.gov Identifier: NCT01581970) to assess the effectiveness of weekly cetuximab associated with twice daily low dose oral cyclophosphamide for 12 weeks, in patients with metastatic HNSCC who have progressed on first line CHT. Primary outcome measure is progression free survival at 2 years. This study is ongoing, but not recruiting participants.

### Consideration

Despite literature data are still restricted and thus definitive consideration cannot be drawn, all clinical trials demonstrated that MC is a well-tolerated treatment. Severe toxicity was not reported. The full impact of MC on tumorogenesis and prognosis has yet to be realized. The potentiality of MC as RT or CHT sensitizer is still unknown, but its anticancer effect, as well as its lower toxicity profile, can suggest a role in both palliative and consolidation approaches.

When a treatment strategy can yield a good tumor control with a low toxic profile, other determinants should be taken into account in selecting patient subsets, including both patient (quality of life, preference and convenience) and research profile (resource cost, rationale and end-points). Thus MC should represent a convenient opportunity in poor PS or terminal stage patients to improve their quality of life by decreasing pain and maintaining daily function, and, in the same time, to control metastatic disease.

On the other hand, although without a strong scientific basis, MC should be considered a valuable option, especially in those patients with a good PS that are reluctant to accept exclusive palliative care [19]. Several preclinical studies and a few small clinical trials have recently reported encouraging results in adjuvant setting. Which is the most effective MC regimen is still difficult to conclude. In Japan and in several other Asiatic countries, MC with Tegafur/Uracil is the first choice, based on empiric data and on the efficacy demonstrated in adjuvant setting for locally advanced stage HNSCC patients [21–23]. Lam et al. reported the first prospective randomized trial in which a trend of better control of distant metastasis was observed in the adjuvant MC group (10 % versus 32 % for the control group) [21]. Nevertheless, there is no unanimous consensus for the use of one MC regimen over the other, due to the small study populations. Literature data suggested that MC treatment strategy is promising in the treatment of HNSCC, but a large-scale randomized trial should be paramount to test the optimal regimen to be used.

Overall, MC has not been yet tested for treatment in combination with RT and it could provide significant clinical benefit and improve quality of life in recurrent or metastatic HNSCC. The effects could be improved by concurrent administration of RT that inhibits the processes of growth and tumor formation. The rationale behind the use of MC is to reduce adverse drug reactions and to target both endothelial cells and tumor cells which are at proliferating stage [24]. MC and RT could have a synergic effect: to suppress more effectively the proliferating endothelial cells in the tumor area, and, on the other hand, to facilitate tumor cells damage by radiation-induced cell death.

## Conclusion

There is still much to be learned in this field, especially with regard to optimization of the proper drugs, dose, schedule, and tumor applications. We hypothesized that MC could have a role in HNSCC treatment and may be best combined to CHT and RT to improve antiangiogenic and anticancer efficacy. Surely, further studies are needed before MC can be successfully integrated into HNSCC clinical practice.
